# Engineered exosome biomedical technologies for precision diagnosis and therapy in orthopedic diseases

**DOI:** 10.3389/fmed.2026.1788658

**Published:** 2026-04-14

**Authors:** Yuansheng Wu, Ruixuan Ma, Hongjiang Jiang

**Affiliations:** 1 Wendeng Orthopedic Hospital of Shandong Province, Weihai, Shandong, China; 2 Shandong University of Traditional Chinese Medicine, Changqing University Science Park, Jinan, Shandong, China

**Keywords:** biomedical technology platform, engineered exosomes, orthopedic diseases, precision diagnostics, targeted delivery

## Abstract

With population aging and sports-related injuries on the rise, the incidence of osteoarthritis, osteoporotic fractures, nonunion bone defects, and bone tumors continues to increase, while conventional pharmacologic and surgical interventions face limitations in target specificity, safety, and cost-effectiveness. Extracellular vesicles, particularly exosomes, are cell-derived nanoscale vesicles that can be engineered via surface ligand/peptide conjugation, membrane protein engineering, and nucleic acid or protein cargo loading to improve targeting, stability, and controlled release. These advances position engineered exosomes as promising platforms for the diagnosis and treatment of orthopedic disorders. Here, we review exosome architecture and biological properties, and systematically summarize extraction, purification, and engineering strategies, alongside their applications to osteoarthritis, osteoporosis, fracture healing, and bone malignancies. Reported therapeutic mechanisms include promotion of osteogenesis and angiogenesis, immunomodulation and anti-inflammatory effects, and regulation of autophagy and apoptosis. Nevertheless, significant barriers remain for clinical translation. To enable routine clinical use, future work should address product heterogeneity, scalable manufacturing, cargo stability, release kinetics, and long-term safety, supported by robust quality control and standardization. Finally, we adopt a technology-centric framework that maps engineering modalities to orthopedic indications and quantifiable performance metrics, outlining the review's methodological route and evidence synthesis approach.

## 1 Introduction

**Figure 1:**
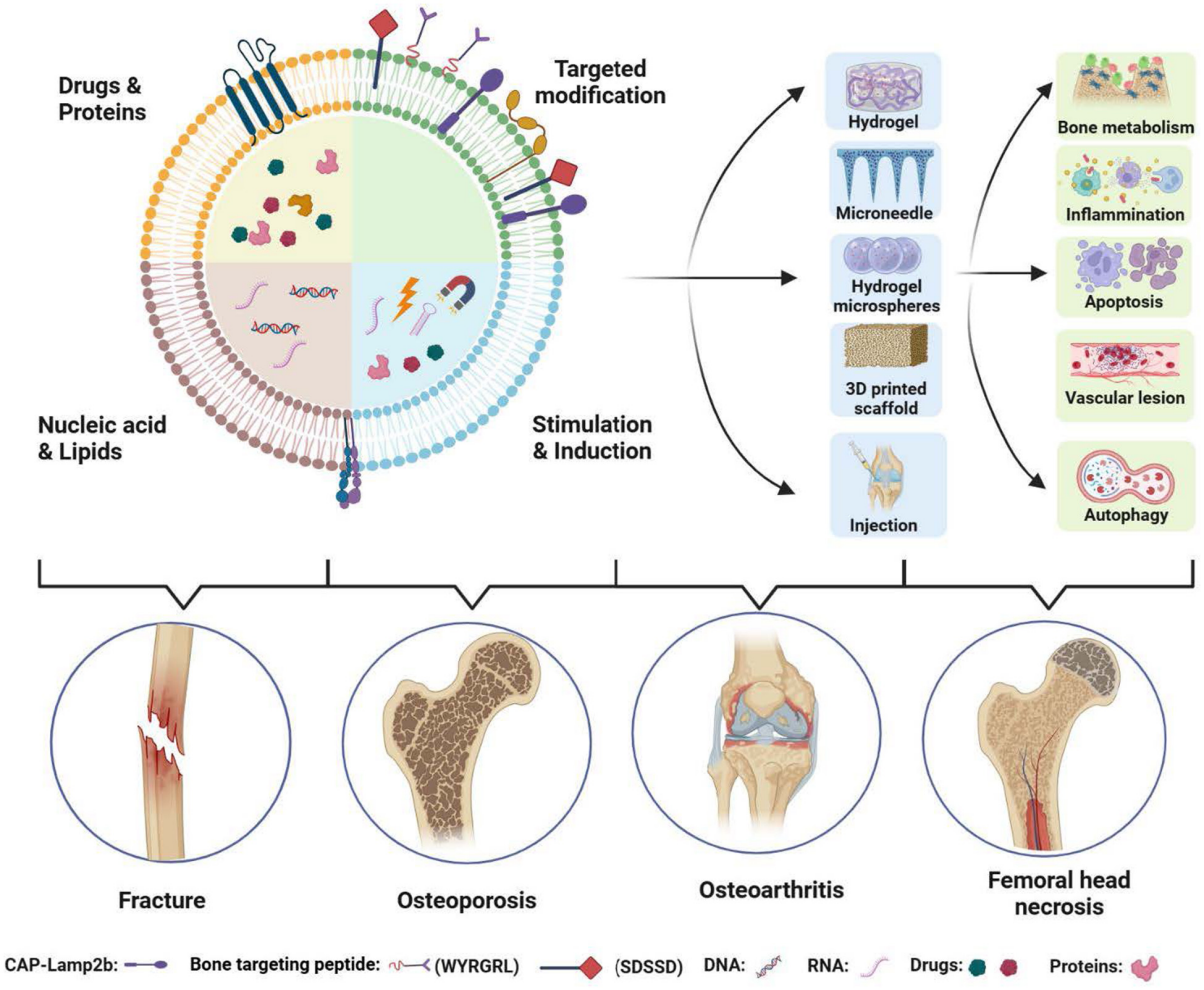
Schematic illustration of engineered exosomes in orthopedic disease therapy. By loading specific therapeutic cargos—such as drugs, genes, and proteins—exosomes can modulate bone metabolism, inflammatory responses, apoptosis, and autophagy, thereby enabling treatment of orthopedic conditions including fracture, osteoarthritis, osteoporosis and ONFH. Their integration with biomaterials, such as hydrogels, microneedle patches, and 3D-printed scaffolds, further broadens their translational applications in clinical settings. Created with BioRender.com. This figure is original and created by the authors.

Extracellular vesicles, particularly exosomes, serve as key mediators of intercellular communication and play a central role in the physiological homeostasis and pathological processes of the skeletal system (1). Carrying a diverse array of bioactive molecules—including proteins and nucleic acids—they directly participate in the regulation of osteogenic differentiation, osteoclastic resorption, inflammatory responses, and angiogenesis. This offers new perspectives for investigating the pathological mechanisms of orthopedic diseases and developing therapeutic strategies (2, 3) (Figure 1). In recent years, engineering modifications have opened novel avenues for enhancing the functionality and precision of exosome-based applications. Through targeted modification and cargo loading of native exosomes, their enrichment capacity and therapeutic efficacy can be significantly improved toward specific pathological sites such as osteoarthritic joints, bone defects, or tumor microenvironments (4, 5). These advances present potential solutions that transcend conventional pharmacological and surgical interventions for major orthopedic conditions lacking ideal treatment options—including osteoarthritis, osteoporosis, refractory bone defects, and bone tumors.

Although current research has accumulated substantial experimental evidence demonstrating the therapeutic potential of engineered exosomes in various orthopedic disease models, these findings remain relatively fragmented. Therefore, it is of significant importance to systematically elucidate the functional principles of engineered exosomes across distinct disease contexts and to establish a coherent logical framework that bridges fundamental discoveries with translational evaluation. This review systematically delineates the therapeutic effects and underlying mechanisms of engineered exosomes in major orthopedic diseases.

The article begins by outlining the biological foundations of exosomes and summarizing the pathological mechanisms of common orthopedic disorders. It then focuses on the regulatory roles of exosomes in key signaling pathways—including bone metabolic balance, apoptosis and autophagy, inflammatory responses, and angiogenesis—while also discussing their potential applications in diagnostics. Finally, the discussion section systematically analyzes the major challenges and future directions for translating exosome-based strategies from laboratory research to clinical practice. This review aims to systematically elucidate the therapeutic effects and underlying mechanisms of engineered exosomes in major orthopedic diseases, hoping to provide reference and insights for their application in orthopedics.

## 2 Exosomes and engineered exosomes

### 2.1 Concept, fundamental structure, and functions of exosomes

**Figure 2:**
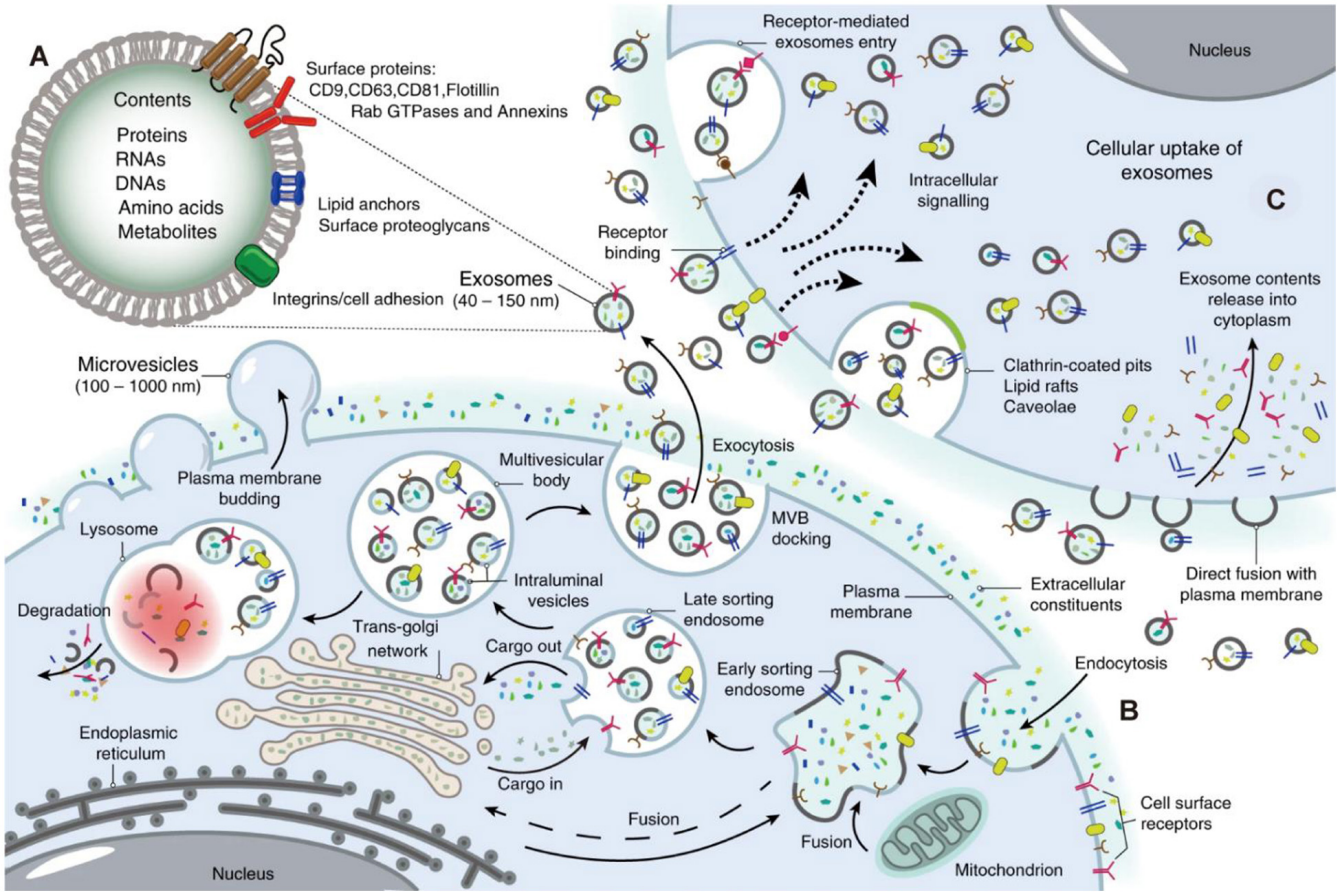
Biogenesis of exosomes. **(A)** Exosomes contain different types of proteins, nucleic acids, amino acids, and metabolites, in which CD9, CD63, CD81, flotillin, and Annexins could be used as markers. **(B)** Extracellular constituents along with cell surface proteins enter cells via the manners of endocytosis and plasma membrane invagination. Plasma membrane bud formation in the luminal side and the fusion of the bud with the constituents of the endoplasmic reticulum (ER), trans-Golgi network (TGN), and mitochondria lead to the formation of early sorting endosomes (ESEs). Then, ESEs give rise to late sorting endosomes (LSEs) in which second invagination via modification of the cargo, leading to the generation of various intraluminal vesicles (ILVs) and the formation of multivesicular body (MVBs). Next, some of MVBs fuse with lysosomes, and the contents in MVBs undergo degradation. Other MVBs can be transported to the plasma membrane and dock on the luminal side of cells. Finally, the exocytosis of MVBs releases ILVs as exosomes to the outside of cells. **(C)** Exosomes enter cells by different manners including fusion with cell plasma membranes, receptor-mediated entry, clathrin-coated pits, lipid rafts and so on (6). Reprinted with permission from Ni et al. (6). Copyright 2020 Springer Nature. Permission obtained for reuse in this publication.

Exosomes are small extracellular vesicles approximately 30–150 nm in diameter. They are released into the extracellular milieu upon fusion of multivesicular bodies with the plasma membrane and are widely present in blood, synovial fluid, and urine. They encapsulate diverse molecular cargos—including proteins, lipids, and RNAs—and efficiently transfer molecular information from one cell to another, thereby playing pivotal roles in multiple physiological processes such as immune regulation, inflammatory responses, and tissue repair (6). Biogenesis proceeds as follows: cells internalize extracellular material via endocytosis to form early sorting endosomes (ESEs). These mature into late sorting endosomes (LSEs), within which the endosomal membrane invaginates to generate intraluminal vesicles, giving rise to multivesicular bodies (MVBs). Finally, MVBs fuse with the plasma membrane to release exosomes into the extracellular space (Figure 2).

Exosomes are bounded by a single phospholipid bilayer into which various surface proteins—such as CD63, CD81, and integrin alpha v beta 5 (αvβ5)—are embedded. These membrane proteins play key roles in mediating interactions between exosomes and target cells (7–10). The intraluminal cargo of exosomes typically includes miRNAs, mRNAs, long non-coding RNAs (lncRNA), proteins, and lipids. Its composition can vary with the identity and physiological state of the donor cell. These components not only mirror features of the cell of origin but also exert diverse biological functions. Studies have shown that exosomes contribute to correcting imbalances in bone metabolism, modulating immune responses, regulating apoptosis, and inducing angiogenesis (2, 3). For example, osteoblast-derived exosomes regulate osteoclast differentiation via the miR-503-3p/Hpse axis and promote post-injury tissue repair (11), whereas exosomes carrying bone morphogenetic protein 2 (BMP-2) enhance repair and regeneration in bone defect models (7, 12). Exosomes are also closely implicated in the pathogenesis and progression of multiple diseases. In patients with osteoporosis, aberrant levels of exosomal miRNAs—such as miR-34a-5p, miR-9-5p, and miR-98-5p—have been observed in plasma (13). Modulating the expression of miRNAs within exosomes can suppress apoptosis, increase bone mass, and slow the progression of osteoporosis (14).

### 2.2 Exosome isolation and purification

Exosome preparation is a pivotal step for biomedical applications. Typically, exosomes are isolated from diverse cell types, including tumor cells, fibroblasts, immune cells, and other secretory cells (15). Current purification strategies generally exploit differences in density, size, and surface biomarker expression (16). Differential ultracentrifugation is the conventional approach but often yields low recovery and limited purity (17). Sucrose density gradient centrifugation affords higher-purity exosomes, yet it is time-consuming and requires costly instrumentation (18). Polymer-based precipitation has also been used; however, reduced solubility in these reagents causes co-precipitation of vesicular and non-vesicular proteins, resulting in comparatively low purity (19). Size-based methods—using filters (20), membranes (21), or acoustic filtration (22)—can effectively remove larger vesicles, debris, and cell fragments from complex mixtures, but they lack specificity for exosomes. By contrast, immunoaffinity capture leverages antibodies against exosomal surface markers (e.g., CD63, CD81, CD9) to improve purity, albeit at the expense of greater procedural complexity and higher cost.

Advances in technology have positioned microfluidics as an emerging approach for exosome isolation, offering high efficiency, speed, and minimal damage, and thereby enabling prospects for large-scale production. For example, microfluidic devices functionalized with anti-CD63 capture antibodies improve the specificity of vesicle capture from serum (23), while ciliated micropillar designs further facilitate vesicle separation and batch-scale processing (24). Reátegui et al. (25) developed a microfluidic platform based on immunoaffinity capture that achieved up to 59% efficiency in isolating tumor-derived exosomes. Overall, immunoaffinity-based microfluidic capture has been successfully applied to exosome isolation from plasma with encouraging results, though it is not yet universally applicable to all exosome subtypes.

### 2.3 Definition of engineered exosomes

Engineered exosomes are exosomes that have been deliberately modified and designed to confer specific functions or targeting capabilities. The core concept is to use molecular and cellular engineering to endow exosomes with defined properties. For example, gene editing can introduce specific tags or receptors into donor cells so that released exosomes precisely target selected tissues or cell types; alternatively, ligands or receptors can be displayed on the vesicle surface to recognize and bind target molecules, enabling targeted therapy (26). Exosomes can also serve as carriers for therapeutic cargos—such as RNAs, proteins, or small-molecule drugs—helping to overcome limitations of conventional delivery systems (1). By integrating cell engineering, nanotechnology, and molecular biology, researchers are developing multifunctional, high-efficiency exosome platforms that advance personalized and precision medicine.

### 2.4 Modifications of engineered exosomes

**Figure 3:**
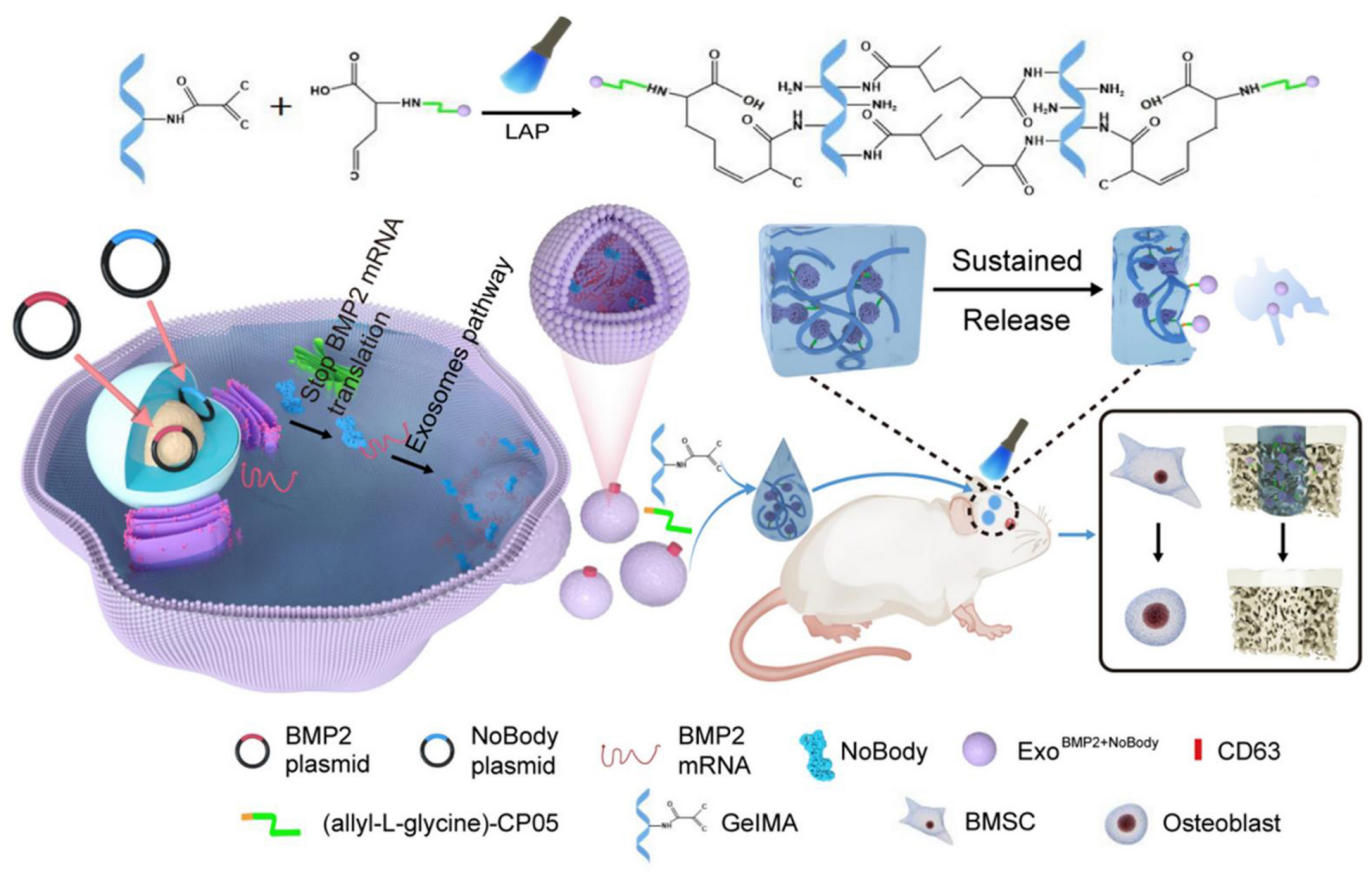
Schematic illustration of CP05 peptide-mediated exosome surface engineering. Reprinted with permission from Yang et al. (7). Copyright 2023 Springer Nature.

Exosome modification during preparation can markedly enhance their performance in drug delivery. Strategies fall into two broad categories: surface engineering and cargo engineering. Surface modifications alter membrane properties to boost targeting and stability. Such modifications not only increase affinity for designated cells but also help mitigate off-target toxicity during delivery. CD63 is a protein enriched on exosome surfaces, and the exosome-anchoring peptide CP05 specifically binds the CD63 antigen. Accordingly, allyl-L-glycine–modified CP05 can form covalent bonds with surface CD63, thereby extending exosome release duration (7) (Figure 3). These proteins serve not only as exosomal markers but also participate in exosome biogenesis, release, and fusion with recipient cells. Chemical crosslinkers that covalently attach targeting moieties (e.g., antibodies, peptides) to the exosomal surface are likewise important surface-engineering approaches. For example, PLGA/PEG-based controlled-release platforms have been developed to achieve programmable exosome release while enhancing their bioactivity (27).

Cargo engineering primarily improves therapeutic efficacy by modulating the intraluminal load. Common approaches include direct drug encapsulation and gene transfection. Direct encapsulation loads drugs (e.g., chemotherapeutics, antibiotics) into exosomes via electrostatic adsorption, hydrophobic interactions, or membrane fusion with liposomes. Gene transfection introduces nucleic acids (e.g., siRNA, mRNA) into donor cells so that secreted exosomes carry specific nucleic-acid therapeutics (28, 29). This strategy is particularly suited to gene therapy applications, for example in the context of COVID-19 vaccines (30).

## 3 Pathological mechanisms of orthopedic diseases

### 3.1 Fractures and impaired bone healing

Critical-sized bone defects caused by trauma, bone tumors, or infection are a frequent challenge in orthopedic practice. The pathophysiology of fracture healing involves multiple biological processes, which become more complex in the presence of a critical defect. Following structural disruption of bone, a local hematoma forms first, providing essential cells and growth factors for subsequent repair. However, the healing cascade can be disrupted by inadequate vascular supply, restricted stem cell differentiation, or excessive inflammatory responses (31). In patients with comorbidities such as diabetes or osteoporosis, both the speed and quality of bone healing are often markedly reduced. Moreover, critical-sized defects are frequently accompanied by persistent inflammation, which leads to growth factor dysregulation and may trigger excessive fibrosis or chondrogenesis, ultimately impeding new bone formation (32). Mechanical instability and aberrant immune responses can further exacerbate healing impairment, resulting in nonunion or prolonged failure of repair. In sum, fracture pathophysiology reflects an interplay of multifactorial insults, necessitating interventions that optimize perfusion, modulate stem cell fate, and control inflammation to improve outcomes.

### 3.2 Osteoarthritis

Osteoarthritis (OA) is a chronic joint disorder caused by the progressive degeneration and destruction of articular cartilage. It is typically triggered by excessive mechanical loading, trauma, genetic predisposition, or immune dysregulation (33). The degeneration of articular cartilage exposes the underlying bone surfaces, leading to synovial inflammation, osteophyte formation, and loss of joint function. Aberrant activation of intra-articular cytokines (e.g., IL-1 and TNF-α) and matrix metalloproteinases (MMPs) accelerates degradation of the cartilage extracellular matrix while suppressing the intrinsic reparative capacity of cartilage (34). Concurrently, sustained inflammation induces synovial thickening (hyperplasia), resulting in further joint damage and pain. Across both conditions, shared pathogenic mechanisms—including inflammatory responses, cytokine activity, alterations in vascular supply, and limited regenerative capacity of resident cells—are key determinants of disease progression (35).

### 3.3 Osteoporosis

Osteoporosis is characterized by reduced bone mass and disruption of bone microarchitecture, resulting in skeletal fragility and an increased risk of fracture (36). Its pathogenesis is multifactorial, with a central feature being an imbalance between bone resorption and bone formation during bone remodeling. Bone remodeling is the coupled process mediated by bone-resorbing osteoclasts and bone-forming osteoblasts within the skeletal tissue (37). In patients with osteoporosis, increased osteoclast activity and/or impaired osteoblast function drive bone resorption to exceed bone formation, leading to a decline in bone mineral density (38). Hormonal changes are closely implicated. For example, postmenopausal declines in estrogen—an inhibitor of osteoclast activity and a promoter of osteoblast function—accelerate bone resorption (39). Other regulators, including parathyroid hormone (PTH), vitamin D, and calcium, also modulate bone metabolism; disturbances in any of these pathways can contribute to the development of osteoporosis (40). In addition, aging slows bone cell turnover and degrades bone microarchitecture, progressively lowering bone density. Overall, osteoporosis arises from the interplay of multiple factors, including dysregulation of bone metabolism, hormonal alterations, lifestyle influences, and genetic susceptibility.

### 3.4 Bone tumors

The pathogenesis of bone tumors involves multiple dimensions, including cellular proliferation, genetic mutations, alterations in the tumor microenvironment, and dysregulated bone metabolism (41). At the cellular level, bone tumor initiation is closely linked to dysfunction of normal skeletal cell populations—particularly aberrant proliferation and differentiation of osteoblasts, osteoclasts, and chondrocytes (42). For example, excessive osteoblastic proliferation can drive abnormal bone formation, whereas hyperactive osteoclasts may lead to excessive bone resorption. In parallel, mutations in key genes—such as p53 and IDH1/2—are major etiologic factors in various bone tumors, including osteosarcoma and chondrosarcoma; these mutations perturb cell-cycle regulation and apoptotic pathways, thereby promoting tumor initiation and progression (43, 44). The tumor microenvironment is another critical determinant. By secreting a spectrum of cytokines and growth factors—such as TGF-β and MMPs—tumor cells remodel local tissue architecture and function, facilitating invasion and metastasis (45). Tumor-associated inflammation and angiogenesis further amplify these processes (46). Overall, the pathogenesis of bone tumors reflects an interwoven, multilevel process encompassing intracellular genetic alterations and signaling dysregulation, together with complex changes in the tumor microenvironment and abnormalities in bone metabolism. The convergence of these factors drives tumor initiation, progression, and dissemination.

### 3.5 Steroid-induced osteonecrosis of the femoral head

Steroid-induced osteonecrosis of the femoral head (SIONFH) is a skeletal disorder associated with prolonged or high-dose exposure to glucocorticoids. Although its pathophysiology is not fully elucidated, it is generally considered to involve multiple contributors, including dysregulated lipid metabolism, microvascular injury, abnormal bone metabolism, and apoptosis (47). Glucocorticoids can induce excessive adipogenesis and marrow fat infiltration, leading to “fat embolism” within the intramedullary space. These fat emboli obstruct the bone microvasculature, reduce perfusion, and precipitate ischemic necrosis of the femoral head (47). Diminished blood flow further compromises oxygen and nutrient delivery, impairs cellular metabolism, and ultimately culminates in death of bone tissue. In addition, steroids directly suppress proliferation and differentiation of bone cells, promoting osteoblast and osteocyte apoptosis and trabecular disintegration, while inhibiting extracellular matrix synthesis, thereby diminishing the intrinsic repair capacity of bone (48). Chronic steroid exposure also suppresses bone formation and enhances bone resorption, producing osteoporosis and increasing fracture risk.

In summary, although orthopedic diseases such as osteoarthritis, osteoporosis, fracture nonunion, bone tumors, and steroid-induced osteonecrosis of the femoral head present with distinct clinical manifestations, their core pathological mechanisms often converge on common pathways, including bone metabolic imbalance, dysregulation of apoptosis and autophagy, inflammatory microenvironment disturbance, and impaired angiogenesis. These pathological features not only serve as key drivers of disease initiation and progression but also provide defined targets for intervention with engineered exosomes. In the following chapter, we adopt a disease-oriented approach to systematically elucidate how engineered exosomes target the specific pathological mechanisms of each condition, exerting therapeutic effects through the regulation of bone homeostasis, inhibition of apoptosis, modulation of autophagy, anti-inflammatory activity, and promotion of angiogenesis.

## 4 Mechanisms of engineered exosomes in orthopedic diseases

Building upon the core pathological features of major orthopedic diseases outlined in Chapter 2, the therapeutic mechanisms of engineered exosomes can be categorized into the regulation of several key biological pathways. It is important to emphasize that different disease contexts determine distinct therapeutic priorities: for instance, osteoporosis and bone defects are dominated by bone metabolic imbalance, osteoarthritis is characterized by chondrocyte apoptosis and synovial inflammation, while osteonecrosis of the femoral head is accompanied by microcirculatory impairment. Therefore, this chapter is organized by disease category, sequentially elaborating on the regulatory roles of engineered exosomes in bone metabolic balance, apoptosis and autophagy, inflammatory responses, and angiogenesis with explicit cross-references to the pathological background established in Chapter 2.

### 4.1 Restoring bone metabolic homeostasis

As discussed in Sections 3.1, 3.3, and 3.5, the core pathological feature of impaired fracture healing, osteoporosis and SIONFH lies in the uncoupling of bone formation and bone resorption. As pivotal mediators of intercellular communication, exosomes have increasingly been recognized as key regulators in correcting bone metabolic imbalance. Bone metabolic homeostasis is maintained by the coupled processes of osteogenesis and osteoclast-mediated resorption, which are tightly governed by diverse signaling molecules. Through their cargo of bioactive molecules, exosomes modulate these cellular activities and thereby influence the dynamic balance of bone metabolism. Evidence indicates that exosomes derived from osteoblasts or bone marrow mesenchymal stem cells (BMSC) promote bone repair and regeneration, with particularly important roles in fracture healing. Exosomes from human adipose-derived mesenchymal stem cells (hAMSC-Exos) enhance the expression of osteogenic proteins such as Runt-related transcription factor 2 (RUNX2) and alkaline phosphatase (ALP) (49), whereas BMSC-derived exosomes (BMSC-Exos) facilitate the maturation of pre-osteoblasts and promote mineral deposition (50). Hypoxia-inducible factor-1α (HIF-1α) is a key transcription factor with dual pro-osteogenic and pro-angiogenic functions in humans (51). HIF-1α-overexpressing BMSC-Exos promote bone regeneration and angiogenesis at defect sites, exhibiting superior bone-repair efficacy compared with conventional BMSC-Exos (52). Macrophages are principal effector cells of the immune response and are intimately connected to bone metabolism (53). Reports indicate that miR-98 carried by exosomes from M1 macrophages suppresses osteogenic differentiation by downregulating dual specificity phosphatase 1 (DUSP1), thereby exacerbating the progression of osteoporosis (54). In contrast, miR-486 within exosomes derived from M2 macrophages promotes osteogenic differentiation via the SMAD2/TGF-β signaling pathway, attenuating the development of osteoporosis (55).

MiRNA can regulate mRNA transcription and protein translation (56). Both age and health status influence the function and expression levels of exosomal miRNA. For example, compared with BMSC-derived exosomes from aged rats, those from young rats display a greater capacity to promote osteogenic differentiation. This is because miR-128-3p in aged rat BMSC-Exos attenuates osteogenesis via SMAD5 signaling (57). Relative to BMSC-Exos from healthy rats, BMSC-Exos from rats with type 2 diabetes mellitus (T2DM) delay the healing of bone defects, attributable to lower miR-140-3p expression in T2DM BMSC-Exos; miR-140-3p promotes bone regeneration through the RhoA/ROCK signaling pathway (58). To date, numerous miRNAs—including miR-26a (59), miR-335 (60), miR-182-5p (61), miR-144-5p (62), and miR-335 (63)—have been implicated in the bone repair process (Table 1).

**Table 1:** The role of exosome-derived ncRNA in bone regeneration.

Origin of exosomes	Nc RNA	Pathway	Up/down	Mechanism	Animal model	References
BMSC	miR-128-3p	Smad5	Down	Inhibit osteoblast differationation	Rat	(57)
BMSC	miR-140-3p	RohA/ROCK	Up	Promot osteogenesis	Rat	(58)
BMSC	miR-26a	—	Up	Promot osteogenesis	Rat	(59)
BMSC	miR-335	Hippo	Up	Promot osteogenesis	Rat	(60)
AB-BMSC	miR-182-5p	PI3K/Akt	Down	Inhibit osteoblast differationation	Mouse	(61)
BMDM	miR-144-5p	Smad1	Down	Inhibit osteoblast differationation	Rat	(62)
BMSC	miR-335	Wnt/β-catenin	Up	Promot osteogenesis	Mouse	(63)
BMDM	miRNA-26a-5p	—	Up	Promot osteogenesis	Rat	(122)
BMSC	miR-21-5p	KLF3	Down	Promote osteogenesis	—	(123)
hBMSC	miR-424-5p	WIF1/Wnt/β-catenin	Down	Inhibitory osteogenesis	—	(124)
BMSC	Hsa_circ_0006859	ROCK1	Up	Inhibitory osteogenesis	Human	(125)
hBMSC	miR-186	Hippo	Up	Promote osteogenesis	Rat	(126)
ADMSC	miR-335-3p	—	—	Promote osteogenesis	Mouse	(127)
Macrophages	miR-486-5p	TGF-β/SMAD2	Up	Promote osteogenesis	Mouse	(55)
Osteoclast	lncRNA AW011738	miR-24-2-5p/TREM1	Down	Inhibitory osteogenesis	Mouse	(68)
Plasma cells	miR-30a-5p	—	—	Inhibitory osteogenesis	Rat	(128)

On the other hand, exosomes also play an important regulatory role in suppressing osteoclast activity. Excessive osteoclast activation is a key pathogenic mechanism underlying disorders such as osteoporosis and osteonecrosis. By interacting with osteoclasts, exosomes modulate their differentiation and functional activity. The HIVEP3 gene encodes Schnurri-3 (SHN3), which suppresses osteogenic differentiation by regulating ERK activity (64), promotes osteoclast activity via RANKL (65), and reduces the formation of type H vessels (66). Accordingly, Cui et al. (67) engineered an exosome-based delivery system for SHN3 siRNA that simultaneously enhanced osteogenic differentiation, inhibited osteoclast activation, and promoted type H vessel formation. In contrast, osteoclast-derived exosomes carrying lncRNA AW011738 inhibited osteogenesis in MC3T3-E1 cells through the lncRNA AW011738/miR-24-2-5p/TREM1 axis, and exacerbated osteoporosis in ovariectomized (OVX) mice (68). Overall, exosomes exert complex, multifaceted effects on bone metabolism. By regulating osteoblast and osteoclast function, shaping local immune responses, and mediating intercellular signaling, they hold considerable potential for maintaining the dynamic homeostasis of bone metabolism.

### 4.2 Regulation of apoptosis

Excessive chondrocyte apoptosis is one of the central pathological events in the progression of osteoarthritis and 2.5 SIONFH (see Sections 3.2 and 3.5). Exosomes can directly or indirectly intervene in the activation of apoptosis-related pathways by delivering specific microRNAs, proteins, and signaling molecule. First, exosomes regulate apoptosis by delivering specific miRNAs that directly modulate the expression of apoptosis-related genes. For example, miR-140 suppresses chondrocyte apoptosis by upregulating anti-apoptotic molecules such as Bcl-2 and Bcl-xL (69). Conversely, some miRNAs (e.g., miR-143 and miR-124) promote apoptosis by upregulating pro-apoptotic gene expression through the NF-κB and ROCK1/TLR9 signaling pathways (70). Platelet-rich plasma (PRP) has provided a non-surgical therapeutic approach for osteoarthritis. Liu et al. (71) reported that PRP-derived exosomes (PRP-Exos) reduce chondrocyte apoptosis via activation of the Wnt/β-catenin pathway, whereas exosomes overexpressing circRNA_0001236 enhance chondrocyte proliferation through the miR-3677-3p/SOX9 axis (72). Moreover, multiple miRNAs—including miR-326 (73), miR-223 (74), miR-338-3p (75), and miR-136-5p (76)—have been implicated in the regulation of chondrocyte apoptosis.

**Figure 4:**
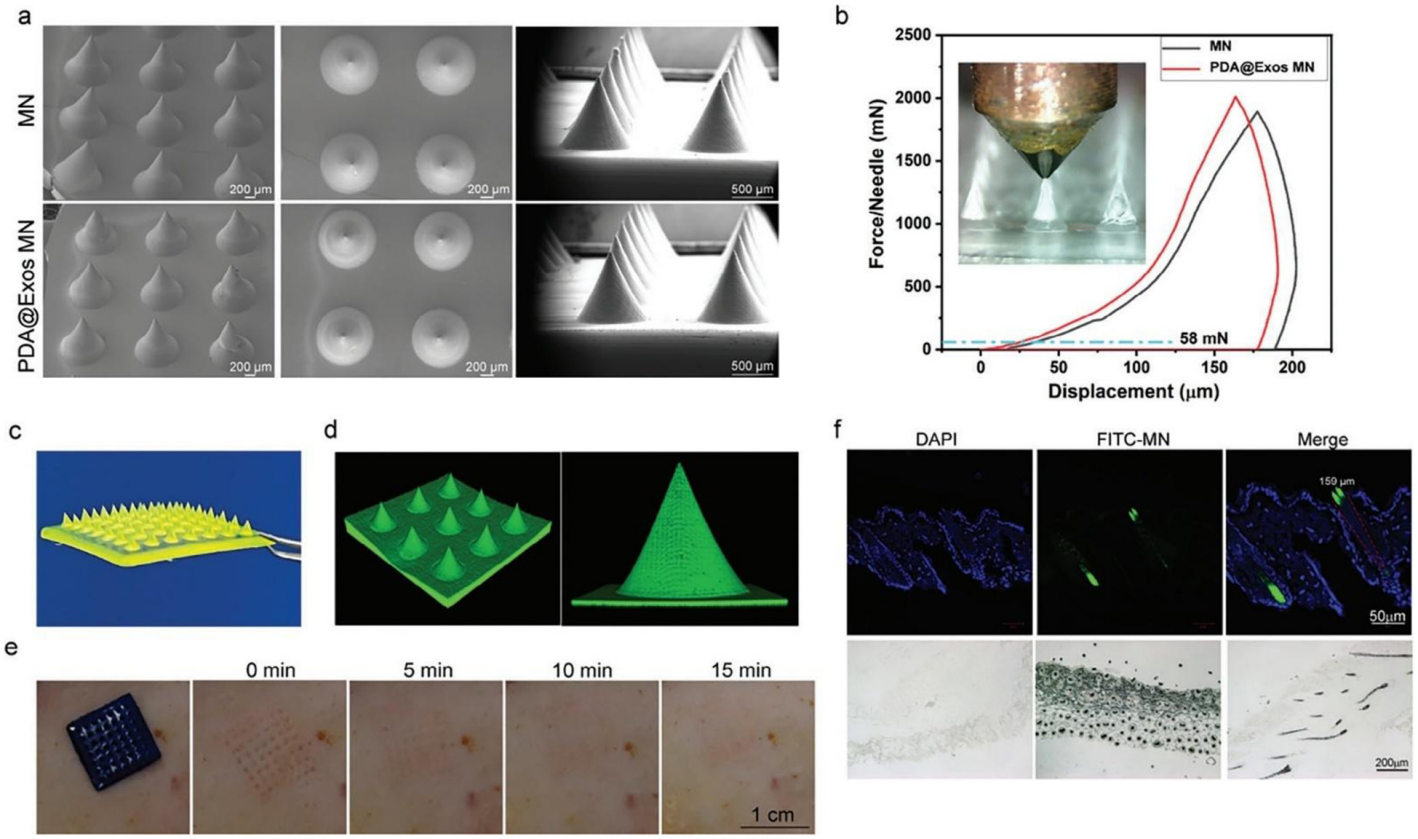
Microstructural Analysis and Mechanical Validation of Microneedles. **(a)** SEM Micrographs of MN and PDA@Exo MN. **(b)** Mechanical Characterization of Microneedles: MN vs. PDA@Exo MN. **(c)** Photomicrograph of a fluorescent MN. **(d)** 3D reconstruction image of Laser Confocal Microscopy Image of a Microneedle. **(e)** Sequential evaluation of skin recovery at various time points post-microneedle insertion. Scale bars: 1 cm. **(f)** Immunofluorescent and histological staining of MN penetration in rat skin. Scale bars: 50 and 200 μm, respectively. Reprinted with permission from Li et al. (79). Copyright 2024 Wiley-VCH GmbH.

Second, the impact of exosomes on apoptosis is closely associated with their regulation of oxidative stress. Oxidative stress is a major trigger of apoptosis, and reactive oxygen species (ROS) promote chondrocyte apoptosis through mitochondrial damage, alterations in gene expression, and suppression of anti-apoptotic signaling. Studies have shown that exosome-mimetic vesicles derived from human umbilical cord mesenchymal stem cells (hUCMSC-EMVs) markedly downregulate dexamethasone (DEX)–induced MAPK pathway activation and ROS accumulation, thereby reducing DEX-induced apoptosis in osteoblastic MC3T3-E1 cells (77). Similarly, BMSC-Exos alleviate nucleus pulposus cell apoptosis induced by oxidative stress and mechanical compression (78). Local targeted delivery of dopamine-modified exosomes via microneedles can also inhibit chondrocyte apoptosis and delay OA progression (79) (Figure 4). In addition, exosomes can indirectly influence apoptosis by modulating immune responses. Carrying either immune-activating or immunosuppressive factors, exosomes regulate the local immune microenvironment and thereby affect apoptosis. Exosomes derived from human umbilical cord mesenchymal stem cells (hUCMSCs) have been shown to downregulate pro-apoptotic protein Bax and pro-inflammatory cytokines (TNF-α, IL-1β), while upregulating anti-apoptotic protein Bcl-2 and the anti-inflammatory cytokine IL-10 (80). The crosstalk between immune regulation and apoptosis arises from their shared signaling networks. Zhang et al. (81) demonstrated that BMSC-Exos enriched in microRNA-181c attenuate spinal inflammation and apoptosis by inhibiting PTEN and the NF-κB pathway, mitigating spinal cord injury. Meanwhile, TLR4 can modulate the expression of downstream genes such as MyD88 and TRAF6 and restrain NF-κB nuclear translocation, thereby regulating apoptosis and inflammatory activation (80).

In summary, the mechanisms by which exosomes regulate apoptosis are multifaceted. By carrying miRNAs, proteins, immune mediators, and antioxidant molecules, exosomes directly or indirectly modulate the initiation and progression of apoptosis. Through the regulation of apoptosis-related genes and selective activation or inhibition of signaling pathways, exosomes alter cellular responsiveness to death signals. Consequently, exosomes not only play vital roles in maintaining cellular homeostasis and immune regulation under physiological conditions but also contribute critically to the onset and progression of various orthopedic diseases.

### 4.3 Regulation of autophagy

**Figure 5:**
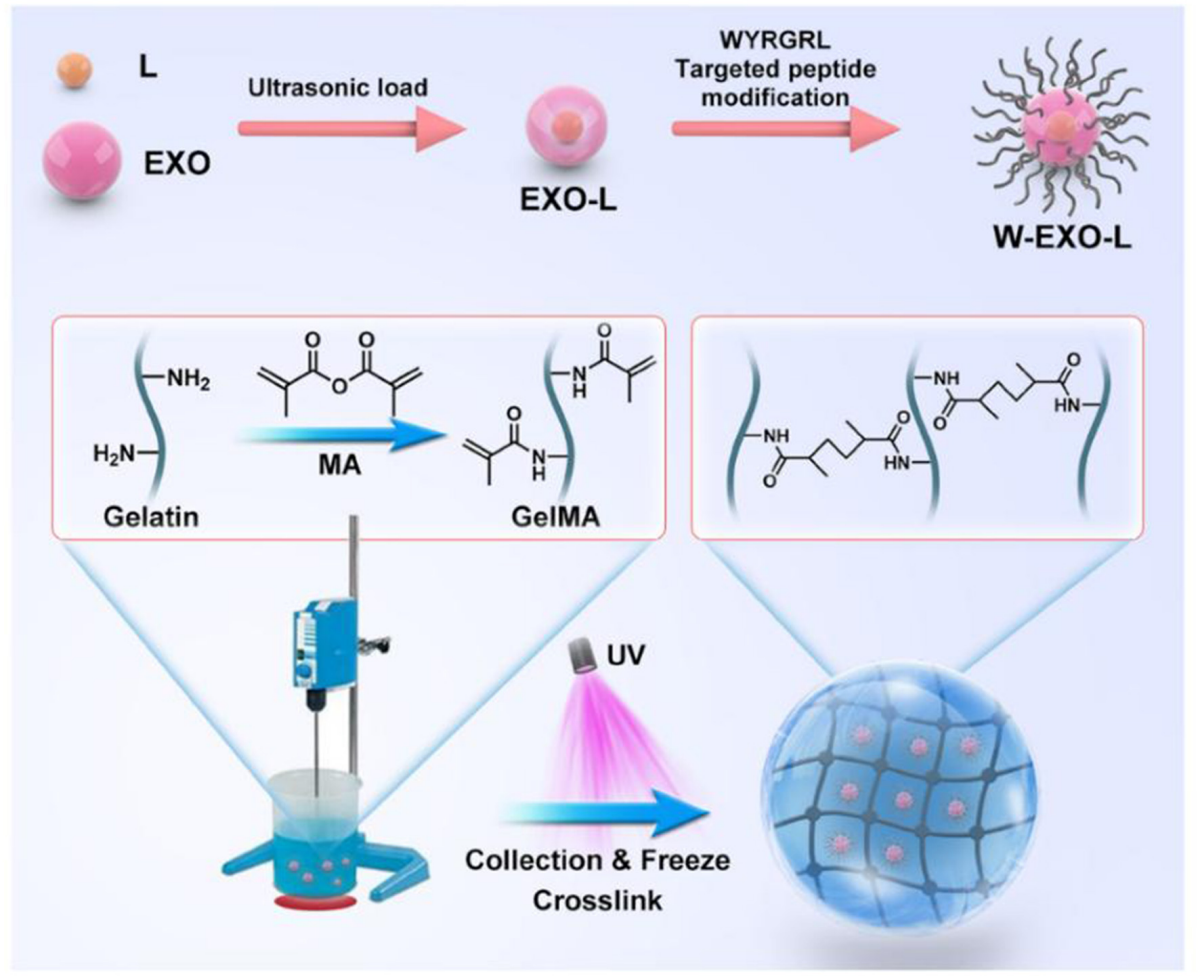
Engineered exosome platform for autophagy regulation in osteoarthritis therapy. The schematic illustrates the construction of WYRGRL peptide-modified exosomes loaded with LRRK2-IN-1 (W-Exo-L) embedded in GelMA hydrogel. This engineering strategy enables targeted delivery and modulation of autophagy-related pathways, as discussed in Section 4.3. The protocol of W-Exo-L@GelMA preparation. L, LRRK2-IN-1; EXO, exosome; W, cartilage affinity WYRGRL (W) peptide; UV, ultraviolet. Adapted with permission from Wan et al. (91). Copyright 2023 Springer Nature.

As discussed in Section 3.2, in addition to osteoarthritis, is also accompanied by dysregulation of autophagy in bone cells. As carriers of intercellular communication, exosomes offer a new perspective on the regulation of autophagy. First, exosomes can modulate the initiation and progression of autophagy by delivering specific molecular cargo. For instance, exosomes with high levels of miR-449a-5p suppress the expression of macrophage autophagy-related genes (e.g., Beclin1), promote inflammasome activation, and thereby inhibit autophagy (82). Conversely, exosome-mediated delivery of long noncoding RNA KLF3-AS1 (83), miR-199a-3p (84), miR-146a-5p (85), and miR-429 (86) enhances chondrocyte autophagy and reduces chondrocyte apoptosis via the PI3K/Akt/mTOR, TRAF6, and FEZ2 signaling pathways (83). Exosomes obtained after TNF-α (87) or fucoidan preconditioning (85) further augment chondrocyte autophagic capacity. Beyond direct pathway regulation, integrating exosomes with biomaterials shows broad translational potential. Hydrogels, nanofibers, and scaffolds can provide stable delivery platforms for exosomes (88, 89). Yin et al. (90) developed a hyaluronic acid–based hydrogel microparticle (HMP) system for exosome delivery, which exhibits favorable injectability and prolongs exosome release. Chemical modification of the exosome surface can additionally improve targeting and expand application scenarios. For example, cartilage-homing WYRGRL peptide–modified exosomes display markedly enhanced targeting, while novel photo-crosslinked spherical gelatin methacryloyl (GelMA) hydrogels further improve exosome bioactivity and release stability (91) (Figure 5).

In addition to direct regulation of autophagy pathways, exosomes can indirectly modulate autophagy by shaping intercellular interactions and the immune milieu. By carrying immunoregulatory factors, exosomes tune immune cell activity, thereby influencing autophagic responses in target cells. Hua et al. (92) reported that exosomes derived from hUCMSCs increase the expression of autophagy-related proteins (LC3-II and Beclin1) via the miR-146a-5p/TRAF6 axis and suppress NLRP3 inflammasome activation in the spinal dorsal horn, alleviating neuroinflammation. Overall, engineered exosomes confer multifaceted protective effects in orthopedic diseases by regulating cellular autophagy, offering new avenues for clinical bone tissue regeneration and therapeutic intervention, and laying a theoretical foundation for exosome carrier design, targeted delivery, and mechanistic studies. Further elucidation of their mechanisms and optimization of engineering strategies will facilitate precision therapeutics and functional recovery in orthopedic disorders.

### 4.4 Regulation of inflammatory responses

Synovial inflammation and sustained activation of pro-inflammatory cytokines within the joint are important drivers of osteoarthritis and delayed bone defect healing (see Sections 3.1 and 3.2). As vehicles of intercellular communication, exosomes deliver diverse bioactive cargos—including proteins, mRNAs, and miRNAs—to modulate immune cell activity and the course of inflammation. For example, exosomes derived from adipose-derived mesenchymal stem cells (AMSC-Exos) suppress inflammation by regulating M1/M2 macrophage polarization via the miR-451a/MIF pathway (93). BMSC-derived exosomes (BMSC-Exos) likewise promote polarization from M1 to M2 macrophages and reduce levels of pro-inflammatory cytokines (94, 95). Low-intensity pulsed ultrasound (LIPUS) further enhances the anti-inflammatory effects of BMSC-Exos (96). By targeting specific genes, miRNAs regulate inflammatory responses and thereby influence joint repair and degeneration. Notably, miR-206 (97) and miR-9-5p (98) within BMSC-Exos suppress inflammatory cytokine expression and inhibit osteoblast apoptosis. BMSC-Exos enriched in the lncRNA TUC339 promote M1-to-M2 polarization, attenuating inflammation and matrix degradation (99). Beyond promoting or suppressing inflammation *per se*, the immunomodulatory actions of exosomes extend to improving tissue repair, maintaining tissue homeostasis, and regulating bone metabolism. When exosomes from M1 or M2 macrophages were co-cultured with BMSC, both enhanced osteogenic differentiation of BMSC, with M1-derived exosomes outperforming those from M2 macrophages (100). In sum, by carrying and transferring multiple bioactive molecules, exosomes modulate immune cell function and the release of inflammatory mediators, thereby shaping the initiation and resolution of inflammation. Their potential in immune tolerance, inflammatory repair, and tissue homeostasis positions exosomes as promising therapeutic targets for inflammatory diseases, offering new clinical avenues.

### 4.5 Influencing angiogenesis

A common pathological basis shared by osteonecrosis of the femoral head and nonunion bone defects is impaired local blood supply (see Sections 3.1 and 3.5). Angiogenesis—the formation of new blood vessels—plays a pivotal role in wound repair and the progression of disorders such as osteoarthritis and ONFH. By transporting diverse bioactive cargos, exosomes regulate key endothelial processes, including proliferation, migration, and tubulogenesis. The most direct pro-angiogenic action of exosomes is to deliver angiogenic factors that activate endothelial cell function. Carrying factors such as vascular endothelial growth factor (VEGF) and fibroblast growth factor (FGF), exosomes can promote endothelial proliferation and migration to facilitate neovessel formation. Notably, rat BMSC-derived exosomes (BMSC-Exos) exhibit strong pro-angiogenic and osteogenic capacities (101). Hypoxia-preconditioned BMSC-Exos further enhance the angiogenic differentiation of HUVECs (102). In addition, hAMSC-derived exosomes (hAMSC-Exos) stimulate endothelial progenitor cell (EPC) proliferation and migration via the NOTCH1/DLL4 signaling pathway, and nanohydroxyapatite/poly-ε-caprolactone (nHA/PCL) scaffolds carrying UC-MSC–derived exosomes (UC-MSC-Exos) accelerate repair of rat calvarial defects (103).

During angiogenesis, exosomal miRNAs regulate endothelial signaling and gene expression to fine-tune vessel formation. For example, miR-100-5p is elevated in serum exosomes from patients with nontraumatic osteonecrosis of the femoral head (NONFH) and suppresses the angiogenic differentiation of HUVECs through the BMPR2/SMAD1/5/9 pathway (104). The immune system, particularly regulatory T cells, is a critical participant in fracture repair (105). Treg-derived exosomes promote angiogenesis via the miR-142-3p/TGFBR1/SMAD2 axis (106). Moreover, several small RNAs, including miR-378 (107), tsRNA-15797 (108), and miR-21-5p (109), show substantial potential to promote endothelial proliferation, migration, and tube formation. Angiogenesis depends not only on endothelial proliferation and migration but also on extracellular matrix (ECM) remodeling. Exosomes modulate angiogenesis by reshaping the vascular ECM. For instance, exosomes carrying specific MMPs—such as MMP2 (110), MMP9 (111), and MMP14 (112)—promote ECM degradation and remodeling, thereby supporting the growth of nascent vessels. In summary, exosomes play multifaceted roles in angiogenesis: they directly regulate endothelial proliferation, migration, and tubulogenesis and indirectly influence vessel formation by modulating immune cell function and the local immune microenvironment. As mechanistic insights deepen, the translational potential of exosomes in wound healing, cancer therapy, and vascular diseases becomes increasingly evident, positioning them as promising therapeutic targets and delivery strategies.

## 5 Exosome-based diagnostics for orthopedic diseases

Exosomes, widely present in blood, synovial fluid, and urine, structurally stable, and enriched with diverse biomolecules, have emerged as promising noninvasive diagnostic biomarkers for orthopedic diseases. Studies show that exosomal profiles of specific miRNAs, circRNAs, and proteins change in patients with orthopedic disorders, reflecting bone metabolic status, the course of bone defects, and degenerative joint pathology. For example, in osteoporotic rat models, exosomal miR-150-3p (14), miR-27a-3p, and miR-196b-5p are downregulated compared with healthy rats (113). Sequencing of plasma exosomal miRNAs in patients with osteoporosis has identified marked differential expression of miR-34a-5p, miR-9-5p, and miR-98-5p (13). In addition, numerous miRNAs—including miR-324-3p, miR-766-3p, miR-1247-5p, miR-330-5p, and miR-3124-5p—show diagnostic potential for postmenopausal osteoporosis (114). However, accurate detection of exosomal miRNAs remains challenging due to their limited abundance in peripheral blood. Luo et al. (115) developed an Enhancing 3D DNA Walker–Induced CRISPR/Cas12a technology that enables highly sensitive detection of exogenous miRNAs associated with osteoporosis.

Early stages of NONFH are often asymptomatic, and the sensitivity of conventional modalities such as CT and MRI is relatively low. Identifying biomarkers for NONFH is therefore critical. In NONFH models, compared with exosomes derived from healthy femoral heads, exosomes from patients exhibited significant differences in the expression of hsa-miR-135b-5p (116), VWF, PRG4 (117), and CD41 (118). RNA sequencing of urine-derived exosomes from patients with hip arthritis revealed 15 miRNAs that were significantly upregulated; among them, hsa-miR-200b-3p and hsa-miR-206 demonstrated diagnostic value (119). Plasminogen activator inhibitor-1 (PAI-1) suppresses the conversion of plasminogen to plasmin, reducing fibrinolysis and increasing the risk of thrombosis (120). Notably, PAI-1 is upregulated in the serum of patients with NONFH but not in those with osteoarthritis, rheumatoid arthritis, or fractures, indicating strong specificity for NONFH diagnosis (121). Looking ahead, by optimizing exosome isolation, labeling, and detection technologies, engineered exosomes hold promise as precision diagnostic platforms for orthopedic diseases, offering safe, noninvasive, and repeatable clinical monitoring.

## 6 Discussion

Engineered exosomes act through coordinated pathways. They promote osteogenesis and angiogenesis, reprogram immunity to counter inflammation, and recalibrate cell-fate decisions by inhibiting apoptosis while enhancing autophagy. Through these multi-target, multi-mechanism actions, they confer programmable therapeutic potential across complex orthopedic microenvironments—including OA, osteoporosis, ONFH, bone defects, and bone tumors. In parallel, exosome-derived biomarkers (miRNAs/circRNAs/proteins) provide molecular-level sensitivity for noninvasive and longitudinal monitoring; when combined with microfluidics and CRISPR-based amplification assays, they offer prospects for earlier and more sensitive detection of chronic orthopedic diseases. To systematically present the logical mapping among “engineering modalities—orthopedic indications—therapeutic mechanisms,” this review synthesizes existing literature to provide a clear methodological reference for researchers in the field (Table 2).

**Table 2:** Technology-function mapping framework of engineered exosomes in orthopedic diseases.

Engineering modality	Specific strategy	Orthopedic indication	Primary functional mechanism	Representative effectors/materials	References
Surface targeting modification	Peptide/antibody conjugation, membrane protein engineering	OA, bone defect, ONFH	Enhanced cartilage/bone targeting, prolonged local retention	WYRGRL peptide, CP05, αvβ5 integrin	(7, 26, 91)
Drug/gene loading	Electroporation, co-incubation, liposome fusion, plasmid transfection	Bone tumor, osteoporosis, bone defect	Delivery of siRNA/miRNA/chemotherapeutic agents, gene expression regulation	SHN3 siRNA, miR-140-3p, doxorubicin	(28, 29, 67)
Preconditioning via cell engineering	Gene editing, hypoxic preconditioning, cytokine induction	Bone defect, OA, ONFH	Enhanced pro-angiogenic/osteogenic capacity, anti-inflammatory effects	HIF-1α, TNF-α preconditioning	(52, 87)
Biomaterial integration	Hydrogel, microneedle patch, 3D-printed scaffold loading	Bone defect, OA, cartilage injury	Controlled release, *in situ* delivery, mechanical support	GelMA, PDA@Exo MN, nHA/PCL	(27, 79, 103)

Despite these advances, several bottlenecks impede translation from laboratory to clinic: (i) product heterogeneity and incompletely defined purity, with no unified characterization metrics (e.g., particle size, cargo composition, functional units) or standardized potency assays; (ii) scalable, reproducible manufacturing still falls short of Good Manufacturing Practice (GMP) and cost-effectiveness requirements, with cargo-loading efficiency and stability during freezing, transportation, and storage requiring further optimization; (iii) *in vivo* biodistribution and pharmacokinetics/pharmacodynamics remain under-quantified; although targeting peptides, membrane-protein engineering, and materials-based controlled release can enhance on-target delivery, immune clearance remains non-negligible; (iv) safety and regulatory pathways are not yet fully defined, including potential risks of immunogenicity and procoagulant/thrombotic events. On the diagnostic side, many candidate biomarkers derive from small, single-center studies; cross-platform assay concordance, establishment of reference intervals, and control of confounders (age, comorbidities, medications) require prospective multicenter validation.

A particular challenge in orthopedic regeneration is the management of critical-sized bone defects (CSDs)—defects that exceed the intrinsic healing capacity of bone. The size threshold for CSDs varies by anatomical location and species, but generally refers to defects larger than 1–2 cm in humans or those resulting in < 10% bone regeneration during the lifetime of the patient. These defects often fail to heal spontaneously due to insufficient vascularization, mechanical instability, and an impaired stem cell niche. Engineered exosomes, when integrated with advanced biomaterials, offer promising new therapeutic options for CSDs. For instance, exosome-loaded 3D-printed scaffolds provide structural support while enabling spatiotemporally controlled release of pro-osteogenic and pro-angiogenic cargo. Hydrogels and microneedle patches functionalized with targeting peptides further enhance local retention and bioactivity. Recent preclinical studies have demonstrated that such combinatorial approaches significantly improve bone volume fraction, vascularization, and mechanical strength in large animal models of CSDs. Nevertheless, the optimal exosome dosage, scaffold degradation kinetics, and synergy with host cells require systematic investigation before clinical translation.

To accelerate clinical translation, we advocate an end-to-end paradigm that is manufacturable, characterizable, and regulatable: (1) Sources and manufacturing: prioritize scalable, lower-risk cell sources (human primary MSCs/immune cells and engineered cell lines), and establish continuous microfluidic separation, large-scale perfusion culture, and in-line quality control; (2) Engineering strategies: implement an integrated design of surface targeting + controlled release + intelligent responsiveness by combining peptide/nanobody guidance, membrane-protein programming, and local delivery platforms such as injectable hydrogels, microneedles/patches, and 3D-printed scaffolds; leverage degradable materials and pH/ROS/enzyme-triggered release to achieve lesion-selective accumulation with appropriate temporal profiles; (3) Safety and regulation: conduct GLP-compliant long-term toxicology and immune/coagulation assessments; establish multidimensional release criteria for residual DNA/RNA, endotoxin, host-cell proteins, and tumor-promoting risk; co-develop exosome-adapted drug–device/drug standards with regulatory agencies; (4) Diagnostic translation: advance multicenter cohorts to lock down biomarker panels, calibrate clinical decision thresholds, and harmonize analytical performance across platforms. In the medium to long term, synergy between exosomes and CRISPR/RNA editing, immune modulation, and osteo-regenerative materials may enable earlier breakthroughs in hard-to-heal bone defects, early ONFH, and recurrent bone tumors. The ultimate goal is to deliver reproducible, affordable, and traceable engineered exosome products that enable a closed loop of precision diagnosis and individualized therapy in orthopedics.

Across representative studies in osteoarthritis, osteoporosis, fracture healing, and bone malignancies, engineered exosome–based approaches improved osteogenesis and angiogenesis readouts (e.g., increased ALP/RUNX2 expression, enhanced micro-CT bone volume fraction and type-H vessel density), reduced inflammatory mediators (e.g., IL-1β, TNF-α), and modulated apoptosis and autophagy pathways (e.g., Bax/Bcl-2 ratio, LC3-II/Beclin1), while exosome-derived biomarkers combined with microfluidics/CRISPR assays enabled earlier and more sensitive detection than conventional approaches, as reported in the cited works. Preclinical safety profiles generally indicated low adverse-event rates. For clinical deployment, a point-of-care diagnostic workflow can integrate venous or synovial-fluid sampling with microfluidic immunocapture and CRISPR-based readout to deliver actionable results within a clinic visit; therapeutically, locally administered peptide-targeted exosomes embedded in injectable hydrogels or applied via microneedle patches for intra-articular delivery—and exosome-loaded 3D-printed scaffolds for critical-sized defects—align with routine orthopedic procedures and enable controlled release.
